# Leiomyomatosis peritonealis disseminata in a nonpregnant woman

**DOI:** 10.4274/tjod.06881

**Published:** 2019-01-09

**Authors:** Abdullah Aydın, Tuçe Söylemez, Ateş Karateke, Mesut Polat, Burçin Rabia Girgin

**Affiliations:** 1 İstanbul Medeniyet University Faculty of Medicine, Department of Pathology, İstanbul, Turkey; 2 İstanbul Medeniyet University Faculty of Medicine, Department of Obstetrics and Gynecology, İstanbul, Turkey

**Keywords:** Nulligravida, leiomyomatosis peritonealis disseminata, abdominal mass

## To the Editor,

**Figure 1:**
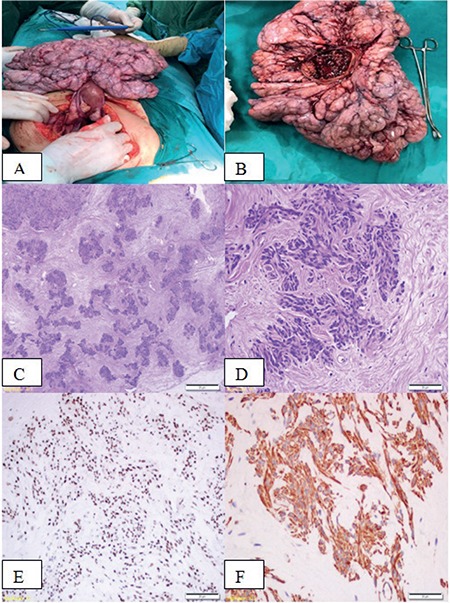
A-F) Intraoperative photography showed multinodular solid mass that had originated from anterior serosal surface of uterus (A, B), microscopic examination revealed smooth muscle proliferation (hematoxylin and eosin Cx40, Dx200), estrogen receptor and caldesmon positivity in tumor cells (E and F x200)

Leiomyomatosis peritonealis disseminata (LPD) is a rare entity characterized by the presence of multiple, small nodules of smooth muscle on the peritoneal and omental surfaces^(1)^. LPD was first described by Wilson and Peale in 1952. Fewer than 140 cases have been reported so far^(1,2)^. It is generally seen in women at reproductive ages; however, there are cases reported in men^(2)^. In most cases, there is an underlying condition such as pregnancy or exogenous hormone replacement, which may cause elevated estrogen levels^(3)^. Here, we present a case of LPD, which developed in a nulligravida woman who received no exogenous hormone therapy. A nulligravida women aged 36 years presented to the gynecology department of İstanbul Göztepe Training and Research Hospital with lower abdominal pain, which she had had for over a year. In her history, there was no history of chronic disease or drug use. She had regular menstrual cycles. In the laboratory evaluation, cancer antigen (CA)-125 was 43.9 U/mL and serum estradiol and progesterone levels were within normal range. A computed tomography scan revealed a mass lesion, which filled the pelvis completely and extended to the peritoneum. Thus, she underwent a laparotomy for removal of the mass lesion. In the laparotomy, a multi-nodular, solid mass lesion originating from the anterior serosal surface of the uterus was seen (Figure 1A, B). Both adnexa were unremarkable. In addition, multiple, solid nodules (2 or 3 cm in size) were present over the peritoneum. There was diffuse, free fluid in abdomen. Mesenchymal neoplasm was primarily considered in the patient who desired to preserve fertility, and therefore a frozen examination was not performed. Fertility was preserved and only excision of masses over the peritoneum and originating from the anterior serosal surface of uterus was performed. Gross pathology showed a multi-nodular mass lesion (30x24x8 cm in size) that consisted of multiple, fibrillary nodules with a creamy-white appearance. On microscopic examination, there were bland smooth muscle cells resembling ordinary leiomyoma without atypia, necrosis or mitosis (Figure 1C, D). Tumor cells were reactive for estrogen receptor, progesterone receptor, desmin, alpha smooth muscle actin, and caldesmon (Figure 1E, F). The Ki-67 index was <1. No S-100, AE1/AE3, cam 5.2 or CD117 expression was observed in the tumor cells. Histopathologic and immunohistochemical findings favored smooth muscle tumor.

LPD is a rare lesion that is mainly seen in young-middle aged women who have hormonal changes due to pregnancy^(2,3)^. Although its pathophysiology is not fully understood, it may be classified in 4 categories including hormonal, iatrogenic, genetic, and subperitoneal mesenchymal cell metaplasia. Although some authors suggest that it arises from subserosal cells with myofibroblastic or smooth muscle cell metaplasia, Travassoli and Norris support the hormonal theory given that most patients are pregnant women or have a history of long-term oral contraceptive use, and that tumor cells express estrogen and progesterone receptors^(4)^. The vast majority of patients are asymptomatic; however, non-specific findings such as abdominal pain, vaginal bleeding or intestinal obstruction caused by the mass lesion may be present in symptomatic patients. We believe that the number of patients with LPD is underestimated in the literature because most cases are asymptomatic^(3)^.

In conclusion, in the differential diagnosis of this entity, peritoneal carcinomatosis and leiomyosarcoma should be considered^(5)^. In our patient, peritoneal carcinomatosis was excluded by histopathologic findings and the absence of primary carcinoma, and leiomyosarcoma was excluded because of the lack of atypia, pleomorphism, necrosis, and high mitotic index^(2)^. LPD is a tumor with benign course in general, although there have been a few cases with malignant transformation^(4,5)^.
